# Perceptions of weight status and energy balance behaviors among patients with non-alcoholic fatty liver disease

**DOI:** 10.1038/s41598-022-09583-1

**Published:** 2022-04-05

**Authors:** Natalia I. Heredia, Ruchi Gaba, Yan Liu, Shilpa Jain, Manasi Rungta, Manav Rungta, Hashem B. El-Serag, Fasiha Kanwal, Aaron P. Thrift, Maya Balakrishnan

**Affiliations:** 1 Department of Health Promotion and Behavioral Sciences, School of Public Health, The University of Texas Health Science Center at Houston, Houston, TX USA; 2 Section of Diabetes, Endocrinology and Metabolism, Department of Internal Medicine, Baylor College of Medicine, Houston, TX USA; 3 Section of Gastroenterology and Hepatology, Department of Internal Medicine, Baylor College of Medicine, One Baylor Plaza, Houston, TX 77030 USA; 4 Texas Medical Center Digestive Diseases Center (DDC), Houston, TX USA; 5 Department of Pathology and Immunology, Baylor College of Medicine, Houston, TX USA; 6 Department of Internal Medicine, Houston VA HSR&D Center for Innovations in Quality, Effectiveness and Safety, Michael E. DeBakey Veterans Affairs Medical Center, Houston, TX USA; 7 Department of Internal Medicine, Baylor College of Medicine, Houston, TX USA; 8 Section of Epidemiology and Population Sciences, Department of Medicine, Baylor College of Medicine, Houston, TX USA; 9 Dan L Duncan Comprehensive Cancer Center, Baylor College of Medicine, Houston, TX USA

**Keywords:** Hepatology, Non-alcoholic fatty liver disease

## Abstract

Weight loss through behavioral modification is central to treating non-alcoholic fatty liver disease (NAFLD). To achieve this, patients need to accurately self-perceive their health behaviors. We aimed to identify predictors of concordance between self-perception and objective measures of body weight, physical activity (PA) and dietary behaviors. We used data from the Harris County NAFLD Cohort, an ongoing prospective study in a regional safety-net healthcare system. Patients completed self-administered baseline questionnaires on demographics, diet, PA, and self-perceptions. We assessed concordance between actual and self-perceived body weight and energy-balance behaviors. Multivariable logistic regression identified predictors of concordance. Patients (n = 458; average age 46.5 years) were 90% Hispanic and 76% female. PA and fruit/vegetable intake guidelines were met among 37% and 9%, respectively. Most (89%) overweight/obese patients accurately perceived themselves as such. However, 41% of insufficiently-active and 34% of patients not meeting fruit/vegetable intake guidelines inaccurately self-perceived their behaviors as “just right”. Women were 3 times more likely to accurately self-perceive weight status (adjusted odds ratio [AOR] 3.24; 95% CI 1.68–6.25) but 51% less likely to accurately self-perceive PA levels than men (AOR 0.49; 95% CI 0.29–0.81). Lower acculturation was associated with higher odds of accurate PA self-perception. Patients with prediabetes or diabetes vs normoglycemia were more likely to accurately self-perceive their fruit/vegetable intake. Most NAFLD patients accurately self-perceived their body weight. A third or more of those not meeting fruit/vegetable intake or PA guidelines had inaccurate perceptions about their behaviors. Our findings highlight key areas to target in NAFLD-specific behavioral modification programs.

## Introduction

Non-alcoholic fatty liver disease (NAFLD) is a significant public health concern in the U.S. It is the second leading indication for liver transplantation^1^, a rising cause for hepatocellular carcinoma^2,3^, and associated with adverse cardiovascular outcomes^4^. Excess body fat is the central pathogenic mechanism underlying NAFLD^5^. Its prevalence has grown rapidly in parallel with obesity, to a current estimate of 25%^6^. Hispanics have a 57% higher risk of NAFLD than non-Hispanic Whites (NHW) and women have a higher risk of advanced NAFLD fibrosis than men^7^.

Behavioral modification with the goal of weight loss through changes in diet and physical activity (PA) is central to NAFLD treatment^8–10^. Weight loss of 5%, 7%, and 10% from baseline is associated with meaningful reductions in hepatic steatosis, steatohepatitis, and hepatic fibrosis, respectively^9^. Reductions in total calorie and sugar intake, increased fruit/vegetable intake and increased aerobic PA are each independently associated with reductions in liver fat and improvements in insulin resistance^8^. As such, clinical guidelines emphasize weight loss, dietary changes, and increased PA^11^.

Behavioral change is a complex process, influenced by multiple cognitive and social determinants. A key initial step toward achieving behavioral change, according to the Transtheoretical Model of Behavior Change^12^, is for patients to transition from having no intention to change to intending to take action through self-reflection and personal reassessment. Patients must believe it is necessary to lose weight and modify their behaviors. While several additional factors ultimately influence the likelihood of behavioral change success, this belief is paramount; without it, patients they are less likely to take action^13^. Thus, an important aspect to assisting patients achieve successful behavior change is to understand if patients’ self-perceptions of body weight and lifestyle behaviors align with objective measures. This is particularly important among Hispanic patients who have the highest rates of NAFLD^14^ and also have, irrespective of NAFLD status, greater weight misperception than NHW^15,16^.

This study aimed to identify the demographic and clinical predictors of concordance between objective measures and self-perception of body weight status, PA, and dietary behaviors among a predominantly Hispanic population of patients with NAFLD at their baseline hepatology clinic visit. The overarching purpose was to elucidate the rates and predictors of discordance in behavioral self-perception, which could serve as targets for counseling. Based on prior research conducted among non-NAFLD populations, we hypothesized that there would be differences in discordance rates based on sex, education level, and acculturation status^17–20^. In addition, we performed an exploratory analysis to characterize clinically significant weight loss, as a proxy for behavioral change, by patients’ baseline concordance between objective measures and self-perception of PA and fruit/vegetable intake.

## Methods

### Study design and population

We conducted a cross-sectional study among patients participating in the Harris County NAFLD Cohort (HCNC) from 2015 to 2019. The HCNC is an ongoing prospective cohort study that includes consecutive adult patients with NAFLD referred to a multidisciplinary clinic staffed by both a hepatologist and an endocrinologist within the Harris Health System, the main regional safety-net healthcare system for residents of Houston, Texas. The inclusion criteria were age ≥ 18 years and presence of NAFLD. The exclusions were presence of alternative liver disease etiologies (described below), decompensated cirrhosis, or hepatocellular carcinoma at baseline. The study was approved by the Baylor College of Medicine and Harris Health System institutional review boards. All research procedures were performed in accordance with relevant guidelines and regulations and in accordance with the Declaration of Helsinki. All participants provided written informed consent.

At the baseline visit, patients completed self-administered questionnaires. Subsequently, patients underwent clinical evaluation that included a detailed liver disease and metabolic assessment.

NAFLD was uniformly diagnosed across the patient population using the following criteria: presence of hepatic steatosis detected by abdominal ultrasound performed within 1 year of the baseline visit and exclusion of patients with chronic excessive alcohol consumption (> 14 and > 21 standard drinks/week on average for ≥ 2 years among women and men, respectively)^11^, viral hepatitis, hemochromatosis, Wilson’s disease, cholestasis, autoimmune disease, drug induced liver injury, or medications associated with hepatic steatosis.

All patients received standard of care management of NAFLD which included clinic based lifestyle modification counseling delivered by both the hepatologist and endocrinologist. The counseling emphasized caloric restriction and a dietary pattern aligned with the MyPlate method which encourages a shift to more vegetable and fruit intake, less added sugars, and a shift away from saturated fats and toward mono-unsaturated fats^21^. Counseling also emphasized moderate intensity aerobic physical activity of at least 150 min weekly^22^. In addition, all patients were referred to a dietitian for continued counseling and returned to the clinic for regular care at 3–6 month intervals. Patients enrolled in the study are being followed over time for changes in weight and development of clinically significant liver disease complications (i.e., hepatic decompensation, hepatocellular carcinoma, or death) and changes in weight. We collected the information at baseline, and ascertained changes in weight prospectively.

### Variables and measures

#### Demographic characteristics and clinical parameters

At baseline, we collected demographic characteristics including age, sex, race/ethnicity, education, primary language, country of birth, and if born outside the U.S., age moved to the U.S. Three proxy acculturation variables were used: primary language spoken, country of birth, and duration living in the U.S. We classified prediabetes or type 2 diabetes based on the presence or absence of a pre-existing diagnosis reported by the patient or documented in the electronic medical record.

Current alcohol use was assessed using two items. The first assessed whether a participant currently consumed alcohol. If the response was affirmative, then frequency and quantity of alcohol use over the prior 1 year was assessed using the Alcohol Use Disorders Identification Test-C (AUDIT-C)^23^. Scores of < 3 in women and < 4 in men were classified as non-risky; scores ≥ 3 in women and ≥ 4 in men were classified as risky. Smoking was assessed with the National Health Interview Survey standard single-item measure assessing if a participant had smoked ≥ 100 cigarettes in their lifetime and then classifying patients as never, current or former users.

#### Body weight

Each patient’s weight and height were measured at the baseline visit and used to calculate body mass index (BMI). Patients were classified as underweight/normal weight (BMI < 25 kg/m^2^) or overweight/obese, which included anyone who met World Health Organization Weight Status criteria for overweight to obese class III (i.e. BMI ≥ 25 kg/m^2^)^24^. We assessed weight change among patients who had their weight measured during follow-up clinic visits 9 to 24 months post-baseline (median = 15 months).

#### Physical activity measures

We used the Rapid Assessment of Physical Activity Scale (RAPA), a 7-item questionnaire validated among both Spanish and English speaking samples that asks participants how physically active they are^25,26^. Using standard scoring procedures, participants were classified as active or insufficiently active. Active was defined as moderate-intensity PA for ≥ 30 min/day for ≥ 5 days/week or vigorous-intensity PA for ≥ 20 min/day for ≥ 3 days/week per American College of Sports Medicine guidelines^27^; insufficiently active was defined as all other responses.

#### Dietary variables

We used the modified version of the National Cancer Institute’s FLASHE screener (26 items) to capture daily intake of dietary factors commonly consumed in the U.S.^28^, including fruits, vegetables, sugar-sweetened beverages (SSB) and sweet foods. Daily fruit/vegetable intake was quantified using two items that assessed the average number of servings consumed each day. Because dietary guidelines specific to NAFLD do not exist, we applied national dietary guidelines that are widely applicable. These indicate that adult women need at least 1½ cups of fruit and 2½ cups of vegetables and adult men need at least 2 cups of fruit and 3½ cups of vegetables daily^29^. We classified patients as either meeting or not meeting these guidelines.

Daily SSB intake was calculated using 4 items capturing frequency of consuming sweetened fruit drinks and teas, regular soda/pop, sports drinks, and pure fruit juice. Daily sugary food intake was calculated with 4 items capturing frequency of consuming candy or chocolate, sugary baked items, frozen desserts, and sugary cereals. We converted responses based on frequency to average daily intake and summed the 4 relevant items to determine daily SSB and sugary food intake^30^.

#### Self-perceptions of body weight, dietary patterns, and exercise behaviors

At baseline, a series of single-items were used to assess how patients perceived their own body-weight status, dietary patterns and PA. To assess self-perception of weight status, participants were asked whether they considered themselves to be “overweight” versus “underweight or about right”^31–34^. Using yes/no responses, participants were also asked if they felt like they needed to lose weight, thought that they should eat healthier, considered themselves to be physically activity, and wanted to be more physically active. Three items assessed whether participants thought they ate the right amount of or needed to eat 1) more fruits/vegetables, 2) less fat, and 3) less sugar^35^.

### Statistical analyses

We used descriptive statistics to assess demographic and behavioral characteristics and tested sex differences. Baseline concordance was calculated between (1) measured BMI vs. participant perception of being overweight/obese or underweight/about right, (2) fruit/vegetable intake measured by the FLASHE dietary screener vs. participant perception of eating the right amount or needing to eat more fruits and vegetables, and (3) PA level measured by the RAPA vs. participant perception of being active or not. We compared the demographic and clinical characteristics between people with discordant versus concordant perceptions of weight status, fruit/vegetable intake, and physical activity status using chi square analysis and student t test.

Univariable and multivariable analyses assessed the cross-sectional association of demographic and clinical characteristics with accuracy of (1) weight status, (2) PA levels and (3) fruit/vegetable intake. The multivariable models included all characteristics from univariable analysis.

Lastly, we explored differences in demographic and clinical characteristics, as well as baseline accuracy of PA and fruit/vegetable intake perceptions between those who did and did not achieve clinically significant weight loss (CSWL) defined as ≥ 5% weight loss one year later. Given the limited sample size and the exploratory nature of this aim, we did not perform tests of statistical significance. All analyses were performed using SAS 9.4 (SAS Institute Inc., Cary, NC, USA).

## Results

The study population’s (n = 458) average age was 46.5 years; 76.0% were women, 90.0% were Hispanic, and 53.5% had diabetes. Overall, 96.5% of the study population were overweight or obese: 21.8% met WHO criteria for overweight status, 35.2% for obesity I, 19.4% obesity II, and 20.1% obesity III (Table 1). Approximately 9.0% of patients met or exceeded the fruit/vegetable intake guidelines and 36.7% were physically active (Table 2). Most reported that they needed to lose weight (88.7%) and wanted to be more physically active (89.7%). While 91.9% reported that they could eat healthier in general, only 60.5% thought that they should eat more fruits/vegetables, 69.2% thought that they should eat less fat and 65.1% thought they should eat less sugar.

**Table 1:** Demographic and clinical characteristics at baseline.

Variable	All participants	Males	Females	p-value
M (SD) or N (%)	N = 458	N = 110	N = 348	
Age, M (SD)	46.45 (11.40)	43.13 (12.23)	47.50 (10.93)	**0.0004**
**Ethnicity**				0.986
Hispanic	412 (89.96)	99 (90.00)	313 (89.94)	
Non-Hispanic	46 (10.04)	11 (10.00)	35 (10.06)	
**Primary language**				0.066
Spanish	350 (76.42)	75 (68.18)	275 (79.02)	
English	76 (16.59)	24 (21.82)	52 (14.94)	
Both Spanish and English	14 (3.06)	7 (6.36)	7 (2.01)	
Other	12 (2.62)	3 (2.73)	9 (2.59)	
Missing	6 (1.31)	1 (0.91)	5 (1.44)	
**Country of birth**				0.050
United States	69 (15.1)	17 (15.5)	52 (14.9)	
Mexico	256 (55.9)	51 (46.4)	205 (58.9)	
Central America	107 (23.4)	36 (32.7)	71 (20.4)	
Other	26 (5.7)	6 (5.5)	20 (5.8)	
**Length in US (years), M (SD)**	21.74 (10.60)	21.62 (10.87)	21.77 (10.54)	0.914
**Education**				0.088
Less than high school	189 (41.27)	44 (40.00)	145 (41.67)	
Some or completed high school	139 (30.35)	36 (32.73)	103 (29.60)	
More than high school	99 (21.62)	28 (25.45)	71 (20.40)	
Missing	31 (6.77)	2 (1.82)	29 (8.33)	
**Average BMI (kg/m**^**2**^**)**	34.70 (7.22)	34.44 (7.90)	34.79 (7.00)	0.657
**Body mass index categories**				0.874
18.5 to < 25 kg/m^2^	16 (3.49)	5(4.55)	11(3.16)	
25 to < 30 kg/m^2^	100 (21.83)	24(21.82)	76(21.84)	
30 to < 35 kg/m^2^	161 (35.15)	41(37.27)	120(34.48)	
35 to < 40 kg/m^2^	89 (19.43)	21(19.09)	68(19.54)	
≥ 40 kg/m^2^	92 (20.09)	19(17.27)	73(20.98)	
**Diabetes**				0.349
No prediabetes or diabetes	149 (32.53)	42 (38.18)	107 (30.75)	
Diabetes	245 (53.49)	54 (49.09)	191 (54.89)	
Prediabetes	64 (13.97)	14 (12.73)	50 (14.37)	
**Smoking status**				** < 0.0001**
Never	359 (78.38)	64 (58.18)	295 (84.77)	
Current	19 (4.15)	7 (6.36)	12 (3.45)	
Former	71 (15.50)	39 (35.45)	32 (9.20)	
Missing	9 (1.97)	0 (0.00)	9 (2.59)	
**Current alcohol use**				**0.0002**
None	314 (68.56)	65 (59.09)	249 (71.55)	
Non-risky	69 (15.07)	22 (20.00)	47 (13.51)	
Risky	40 (8.73)	19 (17.27)	21 (6.03)	
Missing	35 (7.64)	4 (3.64)	31 (8.91)	

Significant p values are in bold.

*M* mean, *SD* standard deviation, *yr* year.

**Table 2:** Behavioral characteristics and perceptions at baseline.

Variable	All participants	Males	Females	p-value
**M (SD) or N (%)**	N = 458	N = 110	N = 348	
**Dietary intake**				
Sugar sweetened beverage daily intake, mean (SD)	1.32 (1.39)	0.90 (1.09)	1.36 (1.41)	**0.036**
Sugary food daily intake, mean (SD)	1.09 (1.24)	0.68 (0.92)	1.14 (1.27)	**0.017**
Fruit servings, daily mean (SD)	2.44 (1.19)	1.87 (1.23)	2.35 (1.15)	**0.0004**
Vegetable servings, daily mean (SD)	2.3 (1.28)	1.96 (1.37)	2.4 (1.23)	**0.0028**
Total fruits and vegetables, daily mean (SD)	4.92 (2.25)	3.90 (2.52)	5.24 (2.44)	** < 0.0001**
**Energy balance behaviors meeting guideline recommendations**				
**Meeting recommended fruit/vegetable intake**				**0.011**
No	362 (79.04)	94 (85.45)	268 (77.01)	
Yes	41 (8.95)	2 (1.82)	39 (11.21)	
Missing	55 (12.01)	14 (12.73)	41 (11.78)	
**Meeting recommended physical activity levels**				0.801
Insufficiently active	254 (55.46)	58 (52.73)	196 (56.32)	
Active	168 (36.68)	43 (39.09)	125 (35.92)	
Missing	36 (7.86)	9 (8.18)	27 (7.76)	
**Energy balance behavior self-perceptions**				
**Perceived weight status (*Do you consider yourself to be overweight, underweight or about right?*)**				**0.015**
Underweight/About right	57 (12.45)	21 (19.09)	36 (10.34)	
Overweight	357 (77.95)	75 (68.18)	282 (81.03)	
Missing	44 (9.61)	14 (12.73)	30 (8.62)	
**Perceived need for weight loss (*Do you feel you need to lose weight?*)**				0.136
No	35 (7.64)	13 (11.82)	22 (6.32)	
Yes	406 (88.65)	92 (83.64)	314 (90.23)	
Missing	17 (3.71)	5 (4.55)	12 (3.45)	
**Perceived physical activity level (*Do you consider yourself to be physically active?*)**				0.126
Not Active	188 (41.05)	36 (32.73)	152 (43.68)	
Active	233 (50.87)	64 (58.18)	169 (48.56)	
Missing	37 (8.08)	10 (9.09)	27 (7.76)	
**Perceived need to increase physical activity level (*Do you want to be more physically active?*)**				0.816
No	26 (5.68)	7 (6.36)	19 (5.46)	
Yes	411 (89.74)	99 (90.00)	312 (89.66)	
Missing	21 (4.59)	4 (3.64)	17 (4.89)	
**Perceived need to eat healthier (*Do you think you could eat healthier?*)**				0.888
No	12 (2.62)	3 (2.73)	9 (2.59)	
Yes	421 (91.92)	102 (92.73)	319 (91.67)	
Missing	25 (5.46)	5 (4.55)	20 (5.75)	
**Perceived fruit/vegetable intake (*Do you think you eat the right amount of fruits and vegetables now, or do you think you should eat more?*)**				0.565
Should eat more	277 (60.48)	64 (58.18)	213 (61.21)	
Right amount	153 (33.41)	37 (33.64)	116 (33.33)	
Missing	28 (6.11)	9 (8.18)	19 (5.46)	
**Perceived dietary fat intake (*Do you think you eat the right amount of fat in your diet or do you think you should eat less?*)**				0.153
Should eat less	317 (69.21)	78 (70.91)	239 (68.68)	
Right amount	110 (24.02)	21 (19.09)	89 (25.57)	
Missing	31 (6.77)	11 (10.00)	20 (5.75)	
**Perceived sugar intake (*Do you think you eat the right amount of sugar in your diet or do you think you should eat less?*)**				0.28
Should eat less	298 (65.07)	75 (68.18)	223 (64.08)	
Right amount	128 (27.95)	25 (22.73)	103 (29.60)	
Missing	32 (6.99)	10 (9.09)	22 (6.32)	

*M* mean, *SD* standard deviation.

There was high concordance (88.4%) between objective measures and self-perceptions of weight status among the overall study population (Table 3). However, there was less concordance between actual and self-perceived PA levels or fruit/vegetable intake (Table 3). Overall, only 65.8% and 62.8% of all patients concordantly self-perceived their PA levels and fruit/vegetable dietary intake, respectively. Table 4 presents concordance between objective measures and self-perceptions among the patients with the highest risk behavior profiles: 88.5% of overweight/obese, 59.1% of insufficiently active, and 65.8% of those who did not meet fruit/vegetable intake thresholds concordantly self-perceived their status.

**Table 3:** Rates of concordance between actual and self-perceived weight status and energy balance behaviors at baseline in the overall population.

	Underweight/normal	Overweight/obese	Active	Insufficiently active	Meeting fruit/vegetable intake guidelines	Not meeting fruit/vegetable intake guidelines
**All patients**						
Accurate	11 (2.7%)	355 (85.7%)	118 (30.1%)	140 (35.7%)	14 (3.6%)	231 (59.2%)
Inaccurate	2 (0.5%)	46 (11.1%)	37 (9.4%)	97 (24.7%)	25 (6.4%)	120 (30.8%)
**Women**						
Accurate	9 (2.8%)	280 (88.1%)	86 (28.6%)	115 (38.2%)	14 (4.7%)	175 (58.3%)
Inaccurate	2 (0.6%)	27 (8.5%)	30 (10.0%)	70 (23.3%)	23 (7.7%)	88 (29.3%)
**Men**						
Accurate	2 (1.7%)	75 (65.2%)	32 (35.2%)	25 (27.5%)	0	56 (62.2%)
Inaccurate	0	19 (16.5%)	7 (7.7%)	27 (29.7%)	2 (2.2%)	32 (35.6%)

**Table 4:** Rates of concordance between actual and self-perceived weight status and energy balance behaviors at baseline in high risk patients.

	Overweight/obese	Insufficiently active	Not meeting fruit/vegetable intake guidelines
**All patients**			
Total	401	237	351
Accurate	355 (88.5%)	140 (59.1%)	231 (65.8%)
Inaccurate	46 (11.5%)	97 (40.9%)	120 (34.2%)
**Women**			
Total	307	185	263
Accurate	280 (91.2%)	115 (62.2%)	175 (66.5%)
Inaccurate	27 (8.8%)	70 (37.8%)	88 (33.5%)
**Men**			
Total	94	52	88
Accurate	75 (79.8%)	25 (48.1%)	56 (63.6%)
Inaccurate	19 (20.2%)	27 (51.9%)	32 (36.4%)

High risk patients defined as patients with BMI ≥ 25 kg/m^2^, who were insufficiently active, or did not meet minimum recommended daily fruit/vegetable intake.

Table 5 and Supplementary Table S1 present the associations between patient characteristics and accuracy of self-perceptions. In the multivariable model, women were three-fold more likely as men to concordantly perceive their weight status (adjusted OR (AOR) 3.24, 95% CI 1.68–6.25). Hispanic ethnicity was associated with three-fold greater odds of concordantly self-perceiving weight status on univariable analysis (OR 3.04, 95% CI 1.49–6.21); this association was attenuated and no longer statistically significant on multivariable analysis (AOR 1.87, 95% CI 0.54–6.51). Women were 51% less likely than men to concordantly self-perceive their PA levels (AOR 0.49, 95% CI 0.29–0.81). Lower acculturation was also independently associated with 2.5-fold higher odds of concordant self-perceived PA levels: Spanish vs. English/bilingual (AOR 2.55, 95% CI 1.35–4.80). Those with prediabetes or diabetes had 2.6-fold greater odds of concordantly perceiving the adequacy of their fruit/vegetable intake than those with normoglycemia (AOR 2.63, 95% CI 1.61–4.31).

**Table 5:** Associations between patient characteristics and accuracy of perceptions at baseline.

	Univariable (OR (95% CI))	Multivariable (OR (95% CI))
**Accuracy of perceived weight status (n = 414)**		
Age (year)	0.97 (0.95–1.00)	0.98 (0.95–1.00)
Female vs. male	2.20 (1.22–3.99)	**3.24 (1.68–6.25)**
Hispanic vs. non-Hispanic	3.04 (1.49–6.21)	1.87 (0.54–6.51)
**Language**		
Spanish only vs. English/Bilingual	0.89 (0.43–1.84)	0.62 (0.23–1.63)
**Education**		
Some/completed high school vs. Less than high school	1.12 (0.56–2.21)	1.19 (0.54–2.61)
More than high school vs. Less than high school	0.84 (0.41–1.69)	0.79 (0.35–1.75)
Diabetes or prediabetes vs. normoglycemic	1.22 (0.69–2.18)	1.34 (0.71–2.53)
**Accuracy of perceived physical activity level (n = 421)**		
Age (year)	1.02 (1.00–1.03)	1.02 (1.00–1.04)
Female vs. male	0.63 (0.40–1.00)	**0.49 (0.29–0.81)**
hispanic vs. non-hispanic	1.73 (0.91–3.30)	1.26 (0.49–3.21)
**Language**		
Spanish only vs. English/Bilingual	2.74 (1.67–4.50)	**2.55 (1.35–4.80)**
**Education**		
Some/completed high school vs. Less than high school	0.68 (0.42–1.07)	0.97 (0.57–1.62)
More than high school vs. Less than high school	0.54 (0.33–0.90)	0.78 (0.44–1.39)
Diabetes or prediabetes vs. normoglycemic	0.78 (0.51–1.18)	0.69 (0.44–1.08)
**Accuracy of perceived fruit and vegetable intake (n = 430)**		
Age (year)	1.01 (1.00–1.03)	1.00 (0.98–1.02)
Female vs. male	0.94 (0.59–1.49)	0.93 (0.56–1.54)
Hispanic vs. Non-hispanic	0.73 (0.39–1.36)	1.21 (0.47–3.15)
**Language**		
Spanish only vs. English/Bilingual	1.26(0.75–2.09)	1.15 (0.60–2.21)
**Education**		
Some/completed high school vs. Less than high school	0.75 (0.47–1.21)	0.80 (0.47–1.35)
More than high school vs. Less than high school	0.78 (0.46–1.31)	0.88 (0.49–1.57)
Diabetes or prediabetes vs. normoglycemic	2.59 (1.63–4.13)	**2.63 (1.61–4.31)**

*OR* odds ratio, *CI* confidence interval.

Bold numbers indicate p < 0.05.

Among the 322 patients with available follow-up weight data, 51 (16%) experienced clinically significant weight loss (i.e., ≥ 5% of baseline weight) (Table 6). We observed no differences in baseline characteristics between patients who did and did not experience clinically significant weight loss (Supplementary Table S2). Among the 4 PA concordance groups, the highest proportion of clinically significant weight loss was observed among patients who were active at baseline but had a discordant self-perception (i.e., felt they needed more physically activity: 38% of 24 patients). Among the 4 fruit/vegetable intake concordance groups, the highest proportion of clinically significant weight loss was observed among patients who accurately self-perceived their fruit/vegetable intake. Specifically, 25% of 12 patients who met daily fruit/vegetable intake guidelines with concordant self-perceived dietary habits, and 21% of 82 patients who did not meet daily fruit/vegetable intake guidelines with concordant self-perceived dietary habits had ≥ 5% weight loss.

**Table 6:** Weight change from baseline to follow-up.

	**n**	**%**
**Proportion of patients with clinically significant weight change (n = 322)**		
≥ 5% weight change among overall study population	51	16%
≥ 7% weight change among overall study population	25	8%
≥ 10% weight change among overall study population	9	3%
**Proportion of patients with ≥ 5% weight loss by concordance group**		
**Physical activity (n = 278)**		
Active and accurate (n = 85)	13	15%
Insufficiently active and accurate (n = 70)	11	18%
Active and inaccurate (n = 24)	9	38%
Insufficiently active and inaccurate (n = 99)	12	12%
**Fruit/vegetable intake (n = 288)**		
Meeting guidelines and accurate (n = 12)	3	25%
Not meeting guidelines and accurate (n = 82)	17	21%
Meeting guidelines and inaccurate (n = 17)	1	6%
Not meeting guidelines and inaccurate (n = 177)	22	14%

Median follow-up time was 15 months; of the 322 patients with available follow-up weight, 278 had self-perception and physical activity data, and 288 had self-perception and fruit/vegetable intake data.

## Discussion

Behavioral change leading to dietary modifications, increased PA and weight loss, is central to NAFLD treatment. Prior research has shown that there are multiple determinants of behavioral change that need to be targeted to help patients achieve healthy habits and weight loss. Unfortunately, there is a paucity of research characterizing behavioral determinants among patients with NAFLD, which is important for formulating improved strategies. Our study is the first to provide insight into NAFLD patients’ perceptions regarding their own lifestyle behaviors, which is one of many determinants of behavioral change, upon first presentation to a hepatology clinic. Among a predominantly Hispanic population, we found that most felt they needed to lose weight and overweight/obese patients accurately self-classified their weight status. To achieve and sustain weight loss, it is important to change both PA and dietary behaviors^36^. Therefore it is less reassuring that approximately 34% of patients with inadequate fruit/vegetable intake and 41% of inactive patients believed their behaviors as adequate. These high discordance rates highlight a need to correct patients’ perceptions of these behaviors as a first step toward influencing patients’ readiness for change^37^. It is vital that clinicians and healthcare teams who engage in lifestyle counseling understand patients’ self-perceptions of their current behaviors.

Our findings demonstrate that patients with NAFLD need targeted and detailed counseling regarding specific dietary components. Only 10% of the overall study population actually had adequate fruit/vegetable intake. The fact that only 33% of the study population nevertheless felt their fruit/vegetable intake was “just right” coupled with the large proportion of patients who inaccurately perceived themselves as having adequate fruit/vegetable intake points to the importance of targeting this dietary component during dietary counseling sessions. Fruit/vegetable intake is an important component of the Mediterranean diet, which is associated with reductions in liver fat and widely recommended for patients with NAFLD^38,39^. Fruits/vegetables may be associated with lower hepatocellular carcinoma risk and have beneficial effects on insulin resistance^40,41^. It is notable that a baseline diagnosis of pre-diabetes/diabetes was associated with a 2.6-greater likelihood of accurate self-perceived fruit/vegetable intake. Given that prediabetic/diabetic patients are more likely to have discussed diet with a dietician or physician prior to presenting to a NAFLD clinic, this finding may reflect the importance of teaching patients and correcting misperceptions about adequate daily fruit/vegetable intake. Expanded counseling from a dietician, with a potential focus on improving misperceptions and increasing knowledge, may be key to moving NAFLD patients toward behavioral change.

Our findings also demonstrate that a second high-yield area for nutritional targeting is sugar. The Mediterranean diet also emphasizes reductions in added sugars and intake of mono-unsaturated over saturated fats^42,43^. Across the entire study population, 97% expressed that they could eat healthier but only 65% thought that they should eat less sugar. We did not assess the concordance between actual and self-perceived sugar intake because while national guidelines recommend reductions in and specific thresholds for added sugar, they do not provide specific daily intake thresholds for all dietary sugar. However, it is well established that simple sugar and fructose intake are important to avoid as they drive NAFLD pathogenesis through de novo lipogenesis and insulin resistance^44,45^. Given the relatively high sugary food and beverage intake observed across the study population, it is concerning that 28% felt they were eating the “right” amount of sugar and did not identify a need for reduction. Sugar intake, thus, may represent a high-yield area for targeted intervention.

We examined differences in perceptions by various demographic characteristics, finding that there were differences by gender. Given that women have a higher risk of NAFLD progression than men^7^, it is encouraging that women with NAFLD were three times as likely as men to accurately identify their weight status. It is disconcerting, however, that women were less likely to accurately recognize insufficient PA levels. It is also concerning that 20% of overweight/obese men underestimated their weight status. Central obesity is the strongest risk factor for cirrhosis among Hispanic men in Texas^46^, and inaccurate weight status perception among those who are overweight or obese has been associated with fewer attempts to lose weight^15^. The observed gender disparity in self-awareness regarding weight status may reflect differences in body image perceptions among men versus women and warrants further qualitative study^47^. The immediate clinical implication is that discussions on lifestyle modifications among men with NAFLD may need to begin with a greater emphasis on defining what it is to be medically overweight or obese. Discussions among women should define and emphasize PA recommendations.

We also examined differences in perceptions by acculturation. With greater exposure to new lifestyles, diets, beliefs, and environments, Hispanic immigrants to the U.S. become more “acculturated” which is associated with a higher prevalence of NAFLD risk factors and potentially with transformed perceptions of their own lifestyle habits^48^. We found that one proxy of lower acculturation, namely Spanish-language preference, was associated with nearly 3 times higher odds of accurately self-perceiving PA levels. In other words, Hispanics with greater acculturation were more likely to misperceive their low PA levels as being sufficient and vice versa. This finding may reflect a social desirability bias among highly acculturated Hispanics. More highly acculturated Hispanics tend to engage in higher levels of leisure-time PA^49,50^; thus, they may also be more aware of the expectation of performing leisure-time PA in the U.S. and consequently misrepresent engaging in high levels of PA to their healthcare providers. Given this possibility, exercise counseling administered to highly acculturated Hispanic NAFLD patients (i.e., born in the U.S. and/or prefer English) should emphasize the minimum PA recommendations outlined by the U.S. PA Guidelines for Americans and the medical importance of adhering to them.

### Study limitations

To our knowledge, this is the first study to provide insight into the concordance between actual and self-perceived patterns of weight status, PA and fruit/vegetable intake among patients with NAFLD at baseline, prior to receiving specialty care in a hepatology clinic. However, certain limitations must be acknowledged. Because this was a cross-sectional study, casual links between the study variables and concordance between actual and self-perceived behaviors cannot be established. Several survey questions were single items, limiting our ability to capture the entirety of the construct measured. We used validated and well-recognized dietary and PA questionnaires. Nevertheless, as in any study investigating dietary and PA behavior, misclassification, recall, and social desirability biases are potential limitations^51,52^. The use of a monitor-based PA measure, for example a pedometer, may have provided greater objectivity than self-report, but was not feasible. Although we used validated dietary and PA questionnaires, the PA levels and fruit/vegetable intake were also based on self-report and could have overestimated concordance between these exposures and self-perceptions. Limitations in the available data prevented an assessment of associations between prior dietitian counseling encounters and patients’ self-perceptions. The study population was representative of a safety net outpatient hepatology clinic in Texas, and predominantly, immigrant, Hispanic and lower than high school educational attainment and representative. We recognize that these characteristics limit the generalizability of this study’s finding; at the same time, the study population represents a high risk NAFLD population, understudied in the field of NAFLD, and are important to target with improved dietary interventions.

### Future directions

There is a pressing need for more research addressing the determinants of behavioral change in patients with NAFLD; to date, only eight papers on the topic have been published^53–60^. This study was a focused investigation into the rates and predictors of one behavioral determinant- self-perception- among patients with NAFLD, prior to receiving specialty care in a hepatology clinic. Our exploratory analysis describing the relationship between baseline concordance and follow up weight loss, as a proxy for behavioral change, was limited by sample size. However, given the complexity of behavioral change, we do not expect that concordance in self-perception, alone, will be predictive of weight loss. Therefore, future studies should examine additional behavioral determinants—for example self-efficacy, perceived barriers, and/or outcome expectations—in patients with NAFLD and how they associate with behavioral change over time. In addition, more proximal behavioral outcomes, such as dietary change or increases in physical activity, should be assessed.

## Conclusion

In summary, while most patients with NAFLD accurately self-recognize their weight status, approximately one-third do not recognize inadequate dietary or PA behaviors. A diagnosis of NAFLD may represent a catalyst for behavior change among patients who are overweight/obese and/or have features of the metabolic syndrome. Recent international collaborative efforts on renaming NAFLD as metabolic associated fatty liver disease (MAFLD) is potentially a step in this direction by reframing the liver disease as a consequence of systemic metabolic dysfunction^61^. In addition to helping patients better understand their liver disease, promoting weight loss and healthy behaviors among patients with NAFLD/MAFLD requires well-designed interventions. Our findings provide a starting point for assessing patients’ readiness for behavioral change and key areas to target in NAFLD/MAFLD-specific behavioral modification programs.

## Supplementary Information

Supplementary Tables.
