# Supplementary material for: Perceptions of weight status and energy balance behaviors among patients with non-alcoholic fatty liver disease
Source: Sci Rep. 2022 Apr 5;12:5695. doi: 10.1038/s41598-022-09583-1 (PMC8983643; doi:10.1038/s41598-022-09583-1)
Supplement: Supplementary file 1 — Supplementary Tables. [file 41598_2022_9583_MOESM1_ESM.docx]

Supplementary Table 1. Comparison of characteristics by accuracy of perceived weight status, physical activity level, and fruit/vegetable intake.

|  | **Perception of weight status** | | | **Perception of physical activity level** | | | **Perception of fruit/vegetable intake** | | |
| --- | --- | --- | --- | --- | --- | --- | --- | --- | --- |
| **Variable** | **Inaccurate** | **Accurate** | **p-value** | **Inaccurate** | **Accurate** | **p-value** | **Inaccurate** | **Accurate** | **p-value** |
| **M (SD) or N (%)** | **48** | **366** |  | **134** | **258** |  | **145** | **245** |  |
| **Age, M (SD)** | 48.79 (10.53) | 45.61 (11.61) | 0.0722 | 46.66(11.41) | 45.56(11.37) | 0.3653 | 47.55(11.35) | 44.89(11.72) | 0.0292 |
| **Sex** |  |  | 0.0042 |  |  | 0.4656 |  |  | 0.8935 |
| Male | 19(39.58) | 77(21.04) |  | 34(25.37) | 57(22.09) |  | 34(23.45) | 56(22.86) |  |
| Female | 29(60.42) | 289(78.96) |  | 100(74.63) | 201(77.91) |  | 111(76.55) | 189(77.14) |  |
| **Ethnicity** |  |  | 0.0621 |  |  | 0.9086 |  |  | 0.154 |
| Hispanic | 39(81.25) | 330(90.16) |  | 120(89.55) | 232(89.92) |  | 126(86.90) | 224(91.43) |  |
| Non-Hispanic | 9(18.75) | 36(9.84) |  | 14(10.45) | 26(10.08) |  | 19(13.10) | 21(8.57) |  |
| **Primary Language** |  |  | 0.1002 |  |  | 0.8895 |  |  | 0.0612 |
| Spanish | 35(72.92) | 277(75.68) |  | 99(73.88) | 194(75.19) |  | 106(73.10) | 191(77.96) |  |
| English / Bilingual | 9(18.75) | 80(21.86) |  | 29(21.64) | 56(21.71) |  | 31(21.38) | 51(20.82) |  |
| Other | 4(8.33) | 7(1.91) |  | 4(2.99) | 5(1.94) |  | 7(4.83) | 2(0.82) |  |
| Missing |  | 2(0.55) |  | 2(1.49) | 3(1.16) |  | 1(0.69) | 1(0.41) |  |
| **Country of Birth** |  |  | 0.1333 |  |  | 0.5614 |  |  | 0.0562 |
| United States | 7(14.58) | 62(16.94) |  | 21(15.67) | 44(17.05) |  | 28(19.31) | 36(14.69) |  |
| Mexico/ Central America | 35(72.92) | 285(77.87) |  | 104(77.61) | 203(78.68) |  | 105(72.41) | 200(81.63) |  |
| Other | 6(12.50) | 19(5.19) |  | 9(6.72) | 11(4.26) |  | 12(8.28) | 9(3.67) |  |
| **Length in US** |  |  | 0.9477 |  |  | 0.3226 |  |  | 0.8349 |
| 1-15 years | 11(22.92) | 81(22.13) |  | 26(19.40) | 60(23.26) |  | 30(20.69) | 55(22.45) |  |
| 16-24 years | 13 (27.08) | 87 (23.77) |  | 38(28.36) | 52(20.16) |  | 37(25.52) | 58(23.67) |  |
| ≥25 years | 10(20.83) | 86(23.50) |  | 28(20.90) | 58(22.48) |  | 30(20.69) | 58(23.67) |  |
| Unknown | 14(29.17) | 112(30.60) |  | 42(31.34) | 88(34.11) |  | 48(33.10) | 74(30.20) |  |
| **Education** |  |  | 0.7142 |  |  | 0.8138 |  |  | 0.6002 |
| Less than high school | 19(39.58) | 146(39.89) |  | 53(39.55) | 105(40.70) |  | 57(39.31) | 96(39.18) |  |
| Some or completed HS | 13(27.08) | 120(32.79) |  | 38(28.36) | 80(31.01) |  | 41(28.28) | 83(33.88) |  |
| More than high school | 13(27.08) | 84(22.95) |  | 33(24.63) | 59(22.87) |  | 39(26.90) | 54(22.04) |  |
| Missing | 3(6.25) | 16(4.37) |  | 10(7.46) | 14(5.43) |  | 8(5.52) | 12(4.90) |  |
| **Body Mass Index** | --- | --- | --- |  |  | 0.3894 |  |  | 0.3691 |
| <30kg/m^2^ | --- | --- | --- | 37(27.61) | 61(23.64) |  | 39(26.90) | 56(22.86) |  |
| ≤30kg/m^2^ | --- | --- | --- | 97(72.39) | 197(76.36) |  | 106(73.10) | 189(77.14) |  |
| **Diabetes** |  |  | 0.4900 |  |  | 0.7716 |  |  | <.0001 |
| No prediabetes or diabetes | 18(37.50) | 119(32.51) |  | 44(32.84) | 81(31.40) |  | 31(21.38) | 100(40.82) |  |
| Diabetes or prediabetes | 30(62.50) | 247(67.49) |  | 90(67.16) | 177(68.60) |  | 114(78.62) | 145(59.18) |  |
| **Smoking Status** |  |  | 0.3164 |  |  | 0.7595 |  |  | 0.5384 |
| Never | 33(68.75) | 288(78.69) |  | 110(82.09) | 201(77.91) |  | 113(77.93) | 191(77.96) |  |
| Current | 3(6.25) | 15(4.10) |  | 5(3.73) | 12(4.65) |  | 4(2.76) | 12(4.90) |  |
| Former | 11(22.92) | 56(15.30) |  | 18(13.43) | 40(15.50) |  | 25(17.24) | 40(16.33) |  |
| Missing | 1(2.08) | 7(1.91) |  | 1(0.75) | 5(1.94) |  | 3(2.07) | 2(0.82) |  |
| **Current Alcohol Use** |  |  | 0.6663 |  |  | 0.4393 |  |  | 0.3965 |
| None | 32(66.67) | 248(67.76) |  | 90(67.16) | 176(68.22) |  | 102(70.34) | 163(66.53) |  |
| Non-risky | 6(12.50) | 60(16.39) |  | 17(12.69) | 41(15.89) |  | 18(12.41) | 42(17.14) |  |
| Risky | 6(12.50) | 30(8.20) |  | 11(8.21) | 22(8.53) |  | 10(6.90) | 22(8.98) |  |
| Missing | 4(8.33) | 28(7.65) |  | 16(11.94) | 19(7.36) |  | 15(10.34) | 18(7.35) |  |

Supplementary Table 2. Baseline characteristics among with <5% weight loss vs. ≥5% weight loss

|  | **<5% weight loss** | **≥5% weight loss** | **P-value** |
| --- | --- | --- | --- |
| **N** | 271 | 51 |  |
| **Age (year)** | 46.41 (11.11) | 44.92 (10.19) | 0.3749 |
| **Sex** |  |  | 0.6071 |
| Male | 62(22.88) | 10(19.61) |  |
| Female | 209(77.12) | 41(80.39) |  |
| **Ethnicity** |  |  | 0.323 |
| Hispanic | 243(89.67) | 48(94.12) |  |
| Non-Hispanic | 28(10.33) | 3(5.88) |  |
| **Primary Language** |  |  | 0.4531 |
| Spanish | 214(78.97) | 42(82.35) |  |
| English / Bilingual | 51(18.82) | 7(13.73) |  |
| Other | 6(2.21) | 2(3.92) |  |
| **Country of Birth** |  |  | 0.3219 |
| United States | 42(15.50) | 4(7.84) |  |
| Mexico / Central America | 216(79.70) | 44(86.27) |  |
| Other | 13(4.80) | 3(5.88) |  |
| **Length in US** |  |  | 0.3115 |
| 1-15 years | 57(21.03) | 16(31.37) |  |
| 16-24 years | 57(21.03) | 12(23.53) |  |
| ≥25 years | 69(25.46) | 9(17.65) |  |
| Unknown | 88(32.47) | 14(27.45) |  |
| **Education** |  |  | 0.6145 |
| Less than high school | 115(42.44) | 17(33.33) |  |
| Some or completed high school | 86(31.73) | 19(37.25) |  |
| More than high school | 54(19.93) | 11(21.57) |  |
| Missing | 16(5.90) | 4(7.84) |  |
| **Obesity** |  |  | 0.8727 |
| <30kg/m^2^ | 72 (26.57) | 13 (25.49) |  |
| ≥30kg/m^2^ | 199 (73.43) | 38 (74.51) |  |
| **Diabetes** |  |  | 0.7589 |
| No diabetes or prediabetes | 91(33.58) | 16(31.37) |  |
| Diabetes or prediabetes | 180(66.42) | 35(68.63) |  |
| **Smoking Status** |  |  | 0.6922 |
| Never | 213(78.60) | 44(86.27) |  |
| Current | 13(4.80) | 1(1.96) |  |
| Former | 39(14.39) | 6(11.76) |  |
| Missing | 6(2.21) | 0 |  |
| **Current Alcohol Use** |  |  | 0.109 |
| None | 202(74.54) | 37(72.55) |  |
| Non-risky | 43(15.87) | 7(13.73) |  |
| Risky | 24(8.86) | 4(7.84) |  |
| Missing | 2(0.74) | 3(5.88) |  |
